# The Link between Depression and Chronic Pain: Neural Mechanisms in the Brain

**DOI:** 10.1155/2017/9724371

**Published:** 2017-06-19

**Authors:** Jiyao Sheng, Shui Liu, Yicun Wang, Ranji Cui, Xuewen Zhang

**Affiliations:** ^1^Jilin Provincial Key Laboratory on Molecular and Chemical Genetic, The Second Hospital of Jilin University, 218 Ziqiang Street, Changchun 130041, China; ^2^Department of Genetics and Comprehensive Cancer Center, University of Alabama at Birmingham, Birmingham, AL 35294, USA

## Abstract

Chronic pain, as a stress state, is one of the critical factors for determining depression, and their coexistence tends to further aggravate the severity of both disorders. Unfortunately, their association remains unclear, which creates a bottleneck problem for managing chronic pain-induced depression. In recent years, studies have found considerable overlaps between pain- and depression-induced neuroplasticity changes and neurobiological mechanism changes. Such overlaps are vital to facilitating the occurrence and development of chronic pain and chronic pain-induced depression. In this review, we summarized the role of neuroplasticity in the occurrence and development of the two disorders in question and explored individualized application strategies of analgesic drugs and antidepressants that have different pharmacological effects in the treatment of chronic pain-induced depression. Therefore, this review may provide new insights into the understanding of association between chronic pain and depression.

## 1. Introduction

Chronic pain is usually defined as any persistent or intermittent pain that lasts more than 3 months, which can be categorized along a variety of dimensions, including one of the most important divisions, neuropathic versus nociceptive pain [1, 2]. Neuropathic pain is induced by a lesion or disease involving the nervous system [3], and nociceptive pain occurs as a consequence of actual or threatened damage to nonneural tissue [4]. Chronic pain is a major public health problem, with epidemiological studies reporting that in the USA and Europe, approximately one fifth of the general population are affected [5]. Additionally, as one of the most common and disabling mental disorders, depression has been reported to be the third leading contributor to the global disease burden [6, 7]. Clinical studies have revealed that chronic pain, as a stress state, often induced depression [8–10] and that up to 85% of patients with chronic pain are affected by severe depression [11, 12]. Patients suffering from chronic pain-induced depression exhibit a poorer prognosis than those with chronic pain only; and chronic pain and depression are closely correlated in terms of occurrence and development and are able to mutually promote their own severity progress [13].

To date, neither the corresponding pathophysiological mechanisms of chronic pain and depression nor their mutual correlation has been identified, which poses a huge challenge for the treatment of pain accompanied by depression. However, in recent years, studies have revealed considerable overlaps between pain- and depression-induced neuroplasticity changes and neurobiological mechanism changes. Such overlaps are vital to facilitating the occurrence and development of chronic pain-induced depression. In particular, injury sensory pathways of body pains have been shown to share the same brain regions involved in mood management, including the insular cortex, prefrontal cortex, anterior cingulate, thalamus, hippocampus, and amygdala, which form a histological structural foundation for the coexistence of pain and depression [14]. Furthermore, the volumes of the prefrontal cortex (PFC) and hippocampus have been reported in many studies to be significantly smaller in depressed patients and to be closely related to depression severity [15–17]. In addition, individuals with depression in postmortem studies have also been observed to have a significantly reduced number of PFC synapses, which thus decreases synaptic functions [18]. Meanwhile, the effect of PFC on pain development via the nucleus accumbens has also been verified [19], thus indicating that the occurrence and development of pain and depression may be associated with some identical neuroplasticity changes. Furthermore, maladaptive plasticity changes, which refer to the plasticity in the nervous system that leads to a disruption of the function and may be considered a disease state, have also been indicated in a large number of clinical trials and animal studies [20]. Additionally, these maladaptive plasticity changes may also occur in sensory conduction pathways from the peripheral to the central nervous system and participate in the occurrence, development, and maintenance of chronic pain [3]. In summary, chronic pain and depression may be based on common neuroplasticity mechanism changes, which are a potentially important route for the onset and aggravation of chronic pain and depression. Reviewing the role of neuroplasticity in chronic pain and depression, this paper explores the influence of analgesic drugs and antidepressants with different pharmacological effects on neuroplasticity as well as their contribution to individualized application strategies in the treatment of chronic pain-induced depression.

## 2. Molecular Mechanisms Associated with Chronic Pain and Depression-Induced Neural Plasticity Changes

### 2.1. Monoamine Neurotransmitters

Monoamine neurotransmitters, including serotonin (5-HT), dopamine (DA), and norepinephrine (NE), have been studied in molecular mechanisms involved in chronic pain and depression. The classical monoamine hypothesis proposes that depression may occur as a result of decreased availability of monoamine neurotransmitters such as 5-HT and NE in the central nervous system (CNS) [21], which is supported by strong evidence from many studies [22–24]. Monoamine neurotransmitters are also vital to the occurrence and development of pain. Additionally, electrical stimulation either in the periaqueductal gray or in the rostral ventrolateral medulla may elevate NE levels in cerebrospinal fluid and thus achieve an analgesic effect, which in turn can be blocked by spinal adrenergic antagonists [25].

In exploring the common neuroplasticity changes of chronic pain and depression, attention should also be paid to the midbrain dopaminergic system because it exerts an indispensable role in the control of forebrain functions. In fact, chronic pain has been shown to have the potential to significantly damage DA activity in the limbic midbrain area according to a large body of evidence [26]. The reactivity of the DA system in the limbic midbrain area to significant stimuli has been observed in imaging studies to be reduced in patients with chronic pain [27, 28]. In particular, the DA receptor D2, also known as D2R, is a protein that is known to be involved in the occurrence and development of depression [29]. Reduced overall DA levels and significantly lowered D2R expression were found in Sagheddu et al.'s chronic neuropathic pain rat model [30], which provides possible new neuroplasticity targets for the treatment of chronic pain-induced depression.

### 2.2. Brain-Derived Neurotrophic Factor (BDNF)

As a precursor protein, pro-BDNF can be processed into a mature BDNF through intracellular and/or extracellular proteases [31]. BDNF belongs to the family of neurotrophic factors and is not only involved in the signaling pathways of the PFC and hippocampal dentate gyrus together with its receptor tropomyosin receptor kinase B (TrkB) but is also important in regulating neuroplasticity [32, 33]. Aside from decreasing BDNF expression and function in the PFC, the hippocampus, and other depression-related structures, depression has been found to reduce the blood BDNF levels in affected patients [34–36]. The crucial function of BNDF in pain occurrence and development has also been confirmed by extensive studies. In particular, Yajima et al. found that BDNF released from the spinal cord can form signaling pathways by binding to TrkB, thereby activating the expression of spinal protein kinase C in spinal neurons, which can regulate hypersensitivity to pain and further influence the progression of neuropathic pain [37, 38].

### 2.3. Inflammatory Factors

The association between inflammatory factors and the CNS has become increasingly clear in recent decades. The surrounding inflammatory response has been shown to cause pain and depression; thus, inflammatory response-mediated pain may be more strongly associated with depression [39–41]. By affecting depression-related pathophysiological functional areas via the blood-brain barrier, inflammatory signals can induce changes in neurotransmitter metabolism, neuroendocrine function, and neuroplasticity [40]. Additionally, the depressive symptoms of affected patients receiving systemic treatment for malignant melanoma or hepatitis C virus infection with INF-*α* have been found to be aggravated in several studies, where major depressive disorder (MDD) was clinically diagnosed in up to 45% of sufferers [42–45]. Furthermore, a high ratio of plasma kynurenine and tryptophan in patients undergoing IFN-*α* therapy has been shown to predict depression severity [42, 46, 47].

### 2.4. Glutamate and Its Receptor Subtypes

Glutamate functions as one of the main excitatory neurotransmitters in the CNS and exists in synapses throughout the brain [48]. Furthermore, glutamate and its receptor subtypes, N-methyl-D-aspartic acid (NMDA) receptor and *α*-amino-3-hydroxy-5-methyl-4-isoxazolepropionic acid (AMPA) receptor, have been found to be involved in the occurrence and development of chronic pain and depression [49–51]. In the spinal cord, both increased excitatory system activity and the accompanying reduced inhibitory system are known to contribute to central hyperalgesia and to ultimately lead to the progression of pathological pain [52]. Glutamatergic activity can be promoted through the breakdown of efficient inhibition of the actions of glutamate by GABA. GABAergic transmission is excitatory during fetal early development but becomes inhibitory during late pregnancy, which results from the depolarization caused by the dominance of Na-K-Cl-cotransporter-1 (NKCC1) and K-Cl-cotransporter-2 (KCC2), which are proteins that aid in the active transport of sodium, potassium, and chloride into and out of cells during early fetal development. Similarly, the return of NKCC1 and KCC2 levels to those of the immature state under pathological conditions increases the excitability of GABAergic transmission, thereby weakening the inhibitory effect [53, 54]. Low-dose diazepam (an NKCC1 inhibitor) was administered to the unipolar depression genetic model in Flinders Sensitive Line (FSL) rats in Matrisciano et al.'s study, which found significantly increased behavioral response compared with that of the control group. In the FSL rats, dramatically elevated KCC2 expression, especially in their cerebellum, was revealed via Western blotting and immunohistochemical data. These data suggest that spontaneous depression in animals is associated with amplified GABAergic transmission in the CNS as a result of enhanced KCC2 expression [55]. Similarly, glutamate and its receptors have an important function in pain and its chronification as well. Increased sensitivity to pain may be due to the absence of a GABAergic region in the spinal cord, especially in the dorsal horn [40].

In conclusion, neuroplasticity crucially affects the occurrence and development of chronic pain and depression and may involve the same brain structures, neurotransmitters, and signaling pathways. Through exploring the common neuroplasticity changes of these two disorders, new targeted therapeutic drugs should be able to be developed or these disorders' common targets should be able to be identified for precise treatment of chronic pain-induced depression, which will surely contribute to the improvement of life quality and prognosis in patients suffering from these disorders.

## 3. Analgesic Drugs to Treat Chronic Pain-Induced Depression

### 3.1. Opioids

Opioids are the most effective drugs for treating chronic pains. By combining with the opioid receptor to relieve patients' pain, opioids have been widely applied to treat various chronic pains, such as cancer pain, nociceptive pain, and neuropathic pain.

**Figure 1:**
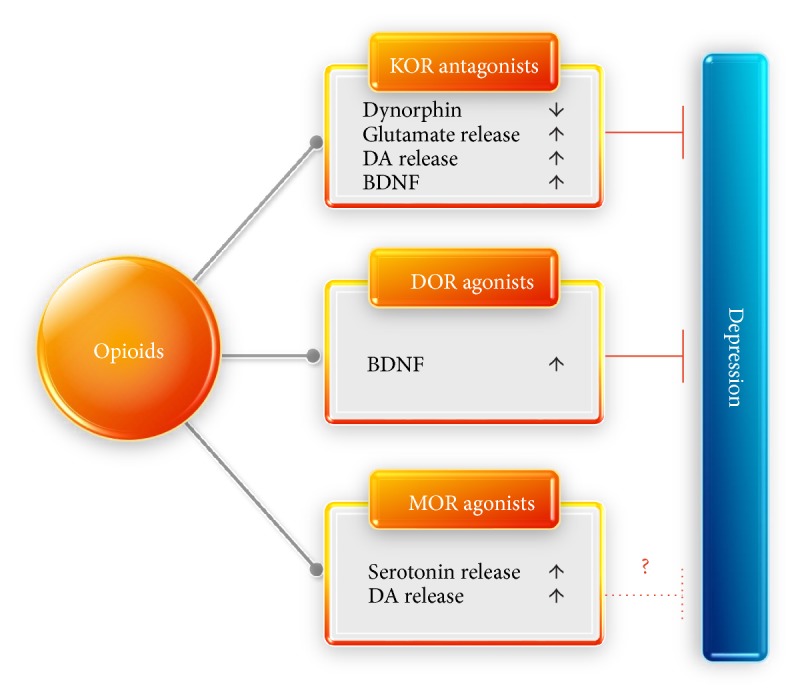
Potential mechanisms of opioids in chronic pain-induced depression therapy.

In recent years, research into the role of opioid receptors in antidepressant therapy has been emerging, with increased concern centered around the potential of opioids in antidepressant therapy. Lots of research suggest that there are three classical types: *μ*, *δ*, and *κ* receptors, all of which involve in regulating mood [56], and some potential mechanisms have been studied (Figure 1) [57–61]. The combined effect of the *μ* receptor agonist and *κ* receptor antagonist was found to have the potential to reduce the occurrence of dysphoria-like behaviors in a study by Tenore [62]. Moreover, the *κ* receptor antagonist has been indicated to have a possibly antidepressant effect in Mague et al.'s animal experimental study [63].

As mentioned earlier, the occurrence and development of depression involve many neurotransmitter systems that are associated with changes in neuroplasticity. The opioid receptor may achieve antidepressant effects by regulating these neurotransmitter systems; this finding has been supported by several studies [56, 64]. Systemic acute morphine injection into mice has been found to have the potential to increase the release of 5-HT in several limbic systems, such as the nucleus accumbens and dorsal striatum. Thus, the finding that the *μ* receptor can achieve an antidepressant effect by controlling the activity of 5-HT neurons is laterally supported [61]. The *κ* receptor is expressed in the nucleus accumbens presynaptically by DA neurons. It can relieve emotions by directly inhibiting the release of DA [65]. Numerous studies shows that *δ* receptor-knockout mice exhibit increased depressive-like behaviors, which indicates that *δ* receptor may become a potential antidepressant target [66–68].

Some animal experiments and clinical studies have demonstrated the effectiveness of some opioids in treating depression [69, 70]. Buprenorphine is a partial agonist of the *μ* receptor and an antagonist of the *κ* receptor and has a good affinity for the *δ* opioid receptor. Because its pharmacokinetics is not influenced by age and renal function, buprenorphine can be used for the middle-aged and the elderly who suffer from refractory depression [71]. A low dosage of buprenorphine for refractory depression in the first 3 weeks was found to significantly reduce depression severity but required long-term maintenance in clinical studies conducted by Karp et al. [70]. The role of buprenorphine in antidepressant therapy, together with samidorphan (an effective *μ* opioid receptor antagonist), was demonstrated via a multicenter, double-blind, and randomized clinical test by Fava et al. [72]. Tramadol is a weak nonopioid agonist of the *μ* receptor and displays properties of TCAs that inhibit the reuptake of 5-HT and NE. Tramadol was found to improve behaviors related to depression and anxiety caused by sciatic nerve injury in Caspani et al.'s examination of a chronic neuropathic pain mouse model [73]. This indicates that certain opioids may enhance synaptic plasticity and achieve the purpose of antidepressant therapy by adjusting neurotransmitter systems.

The potential of opioids in treating chronic pain-induced depression is established; however, application of opioids to antidepressant therapy has been controversial because of patients' severe dependence and addiction to them [74]. The long-term use of opioids has been shown to increase the risk of depression [75] and to even cause hyperalgesia, which can lead to depression [76]. By statistically analyzing the opioid treatment of pain in three independent American health systems, Scherrer et al. found that in the Veterans Health database, patients taking opioids for 31–90 days were found to be at an 18% higher risk for depression than those taking opioids for 1–30 days [77]. Additionally, depression may prolong the duration of opioid use [78]. The duration of opioid use in patients with a history of depression was found to be three times longer than that in patients without depression according to Braden et al.'s study [79]. Therefore, the extensive application of opioids for treating chronic pain-induced depression remains to be further studied and explored.

### 3.2. Benzodiazepines

Benzodiazepines have been demonstrated to have a certain therapeutic effect in treating chronic pains, including neuropathic pain or inflammatory pain [80]. The analgesic mechanism of the benzodiazepines may be associated with the antihyperalgesic effect of the GABA_A_ receptor, which is a molecular target of the benzodiazepines in the spinal cord [81]. Because the GABA_A_ receptors, including the *α*1, *α*2, *α*3, or *α*5 subunit, have also been found to be involved in mood regulation [82], the benzodiazepines have a potential in antidepressant therapy. By studying the GABA_A_ receptor *α*2 subtype homozygous gene-knockout mouse, anxiety and depression-like behaviors of the mouse were markedly increased in the conflict-based novelty-inhibited feeding test and increased in despair-based forced swim test and tail suspension tests in a study by Vollenweider et al. [83]. This result suggests that the benzodiazepines can potentially treat chronic pain-induced depression.

## 4. Antidepressant Drugs for Treating Chronic Pain-Induced Depression

### 4.1. Monoamine Oxidase Inhibitor

Monoamine oxidase (MAO) is an important enzyme in the biogenic amine degradation pathway. MAO can be classified into two types: the type A MAO degrades NE and 5-HT and the type B MAO degrades phenylethylamine and benzydamine [84]; however, evidence suggests that type A MAO is more often implicated in mental disorders, including major depressive disorder [85]. Because clinical depression is associated with a decreased system of NE and/or 5-HT content in some regions of the CNS, the antidepressant effect of the classical monoamine oxidase inhibitor (MAOI) might be related to its ability to increase the NE and/or 5-HT levels of these sites [21, 85]. The mechanism of the classical MAOI is irreversible inhibition of MAO by covalent binding to the active site of the enzyme, but it is nonspecific and can inhibit the liver microsomal enzyme system, which affects the metabolism of many drugs. In addition, some classic MAOI themselves have hepatotoxicity [86], so they are clinically no longer used for treating depression at present. In recent years, the MAOI has been represented by moclobemide, which selectively reversibly inhibits type A MAO and has been attracting attention again due to its side effects. This drug can increase NE, 5-HT, and DA levels in the tissues, which has been confirmed by in vitro and in vivo tests [87]. Its antidepressant effect on the elderly has also been verified by clinical studies [88]. The effect of such drugs on pain treatment has been also confirmed in other clinical studies [89]. Mattia and Coluzzi found that indantadol as an oral and nonselective monoamine oxidase inhibitor and NMDA antagonist had the potential to treat neuropathic pain due to its antihyperalgesic activity [90]. However, the application of monoamine oxidase inhibitors in the treatment of chronic pain-induced depression still requires confirmation through a large number of clinical trials and animal experiments.

### 4.2. Tricyclic Antidepressant Drugs

Tricyclic antidepressant drugs are traditional antidepressant drugs, commonly including amitriptyline, imipramine, nortriptyline, and desipramine. The action mechanism of tricyclic antidepressant drugs may be to first inhibit 5-HT and NE reuptake at the synapse site and then enhance endogenous pain inhibition of the CNS. They are helpful for easing many chronic pains, especially neuropathic pain [91]. Because similar neuroplasticity changes occur during the experience of pain and depression in the monoamine neurotransmitter system, studies focused on the application of tricyclic antidepressant drugs in pain management have emerged unceasingly in recent years. For example, a clinical study by Kopsky and Hesselink indicated that local high doses of amitriptyline were effective for treating neuropathic pain [92]. Furthermore, Rowbotham and colleagues, through a clinical trial of 47 neuropathic pain patients comparing three antidepressants, that is, desipramine, amitriptyline, and fluoxetine, confirmed that all three drugs can reduce pain experienced by postherpetic neuralgia patients, and the tricyclics desipramine and amitriptyline were well tolerated and provided more meaningful pain relief in 53%–80% of all subjects [93].

### 4.3. Monoamine Reuptake Inhibitors

For monoamine neurotransmitters, neurotransmitter reuptake is one of the most important determinants of signal kinetics and regulation of neurotransmitter reuptake also helps regulate the activity of the nervous network across the CNS, thus achieving an antidepressant effect [94]. With more in-depth studies on the treatment mechanism of monoamine neurotransmitters in antidepression and pain relief, inhibitors including selective 5-HT reuptake inhibitors (SSRIs) and 5-HT and NE reuptake inhibitors (SNRIs) have emerged unceasingly and have gradually become first-line drugs for clinical antidepressant therapy [95]. Antidepressant pharmacological mechanisms of SSRIs and SNRIs are to selectively act on some 5-HT and/or NE receptor subtypes and block their reuptake to increase 5-HT and/or NE that are available for biological uses in the synaptic cleft of nerve cells, thus further enhancing monoamine neurotransmission and having an antidepressant effect. The 5-HT and NE reuptake inhibitor antidepressants have been confirmed by numerous studies to be efficacious in chronic neuropathic pain patients [96–98]. Furthermore, average pain relief (recorded via a diary) and maximum pain intensity (retrospective assessment via a computer program) in patients with chronic neuropathic pain were found to be significantly lower with antidepressant venlafaxine, a 5-HT and SNRI, compared with a placebo in Tasmuth et al.'s randomized, double-blind study [99].

### 4.4. Glutamatergic Antidepressant Drugs

As mentioned previously, studies have demonstrated the role of glutamate and its NMDA receptor subtypes in analgesia and antidepressant therapy [100–104]. As a noncompetitive NMDA receptor antagonist, ketamine has been used for anaesthetization since the 1960s and was reported in 2000 to rapidly improve depressive symptoms, including refractory depression within several hours. It has become a new type of antidepressant drug for targeting the glutamatergic system [105]. More importantly, studies have found that ketamine not only increased the number of synaptic connections in the PFC but rapidly improved deficits caused by chronic stress [106, 107]. Furthermore, by antagonizing the glutamatergic NMDA receptor, ketamine was found to accelerate the release of presynaptic glutamate, thereby enhancing the regional activity of the excitatory network, and eventually leading to a significant change in synaptic plasticity and connectivity [108, 109]. This achieves the purpose of analgesia and antidepressant therapy [107]. However, such drugs have side effects such as dizziness, blurred vision, headache, nausea or vomiting, dry mouth, poor coordination, and restlessness [110]. Therefore, the safety and efficacy of ketamine and other NMDA receptor antagonists for treating chronic pain-induced depression remain to be further explored.

### 4.5. Potential Therapy Methods

Although we have summarized clinical drugs that may be therapeutically applied to treat chronic pain-induced depression, their selection for use in therapy is currently still limited. Based on our summary of common neuroplasticity changes in pain and depression, many new therapy targets may provide new future therapy directions for treating chronic pain-induced depression.

The fact that dopaminergic drugs, such as pramipexole, are effective drugs for depression inhibition suggests that enhancing DA function may form at least a partial basis for treatment response of MDD [111]. A change in the function of the DA system in the midbrain margin and the fact that certain antidepressant drugs also had a function in enhancing DA transmission have been demonstrated in studies with rodent depression models [112, 113]. Furthermore, treatment methods for chronic depression including electroconvulsive stimulation, sleep deprivation, and almost all antidepressant drugs have been shown to enhance the role of the DA receptor agonist in motion stimulation [111]. Additionally, data and gene-related animal model studies concluded that DA could relieve pain via the D2 receptor [26]. Also supporting the claim that DA has an analgesic effect, some human studies found an increase in emotional pain rating after DA was exhausted and an improvement in conditional pain after the D2 receptor was activated [114, 115]. Thus, the D2 receptor may serve as a new therapeutic target for chronic pain-induced depression.

The activity-dependent regulation expressed by the early BDNF is associated with neuronal plasticity [31]. The BDNF level of patients with depression has been found to be significantly reduced in several studies [116, 117]. Additionally, a reduced BDNF receptor TrkB level in the brain has also been reported [118]. Activation and phosphorylation of TrkB are also known to be significantly reduced in suicide victims [119, 120]. Furthermore, a decrease in BDNF level can lead to a decrease in hippocampal volume and number of nerves, dendritic reconstruction, loss of glial cells, increasing neurotoxicity, and increasing susceptibility to depression [121]. Meanwhile, the BDNF has been shown to be a crucial signal molecule between microglia and neurons, which is an essential link in neuropathic pain transmission, and blocking this pathway may represent a therapeutic strategy for treating neuropathic pain [122]. Therefore, the BDNF could become a new target for treating chronic pain-induced depression in the near future.

### 4.6. Adjuvant Psychotherapy

In addition to the neural plasticity mechanism described above, psychosocial factors also have a significant effect on the occurrence and development of chronic pain-induced depression [123, 124]. Therefore, appropriate adjuvant psychotherapy also has a crucial role in treating chronic pain-induced depression, which has been confirmed in many clinical investigations [125–127]. For example, by means of randomized clinical trials of 342 patients with chronic back pain between 20 and 70 years of age, the group that received cognitive behavioral therapy versus usual care showed greater improvement in function (adjusted mean difference in range) [128]. Furthermore, Eccleston et al., through a retrospective analysis of 37 clinical randomized trials, found that, for children and adolescents with headache, psychological therapy decreased treatment pain and follow-up pain [129]. Thus, psychotherapy contributes to the relief from clinical symptoms, shortens the duration of the recovery cycle, improves patients' prognosis, and is recommended as a necessary adjuvant therapy for chronic pain-induced depression.

## 5. Summary and Prospects

**Figure 2:**
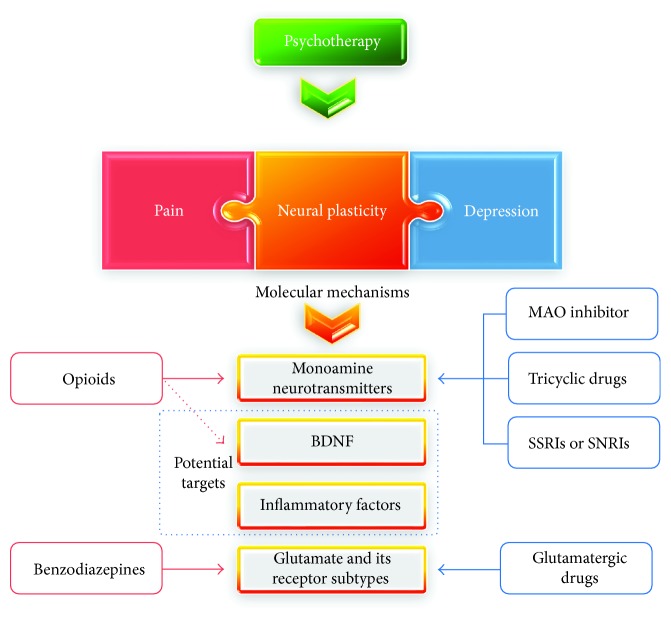
The treatment of chronic pain-induced depression.

In conclusion, pain and depression are closely correlated from the perspectives of both brain regions and the neurological function system, whereby chronic pain may lead to depression. One of the important causes for chronic pain leading to depression appears to be the crucial effect of common neuroplasticity changes on the occurrence and development of the two disorders in question (Figure 2). Nevertheless, current efforts in this field fail to sufficiently and explicitly explain their connection. Further investigations into the common neuroplasticity changes shared by pain and depression are warranted to promote the identification of new drug targets and to free patients from chronic pain-induced depression.
